# Surgical outcomes of the thoracic ossification of ligamentum flavum: a retrospective analysis of 61 cases

**DOI:** 10.1186/s12891-020-03905-y

**Published:** 2021-01-04

**Authors:** Tsuyoshi Yamada, Shigeo Shindo, Toshitaka Yoshii, Shuta Ushio, Kazuo Kusano, Norihiko Miyake, Yoshiyasu Arai, Kazuyuki Otani, Atsushi Okawa, Osamu Nakai

**Affiliations:** 1 Department of Orthopaedic Surgery, Kudanzaka Hospital, 1-6-12 Kudan- minami, Chiyoda-ku, 102-0074 Tokyo, Japan; 2 Department of Orthopaedic Surgery, Graduate School, Tokyo Medical and Dental University, Tokyo, 113-8510 Japan; 3 Department of Orthopaedic Surgery, Saiseikai Kawaguchi General Hospital, 5-11-5 Nishikawaguchi, 332-8558 Kawaguchi-city, Saitama Japan

**Keywords:** Ossification of the ligamentum flavum, Thoracic myelopathy, Tandem spinal stenosis, Lumbar spinal canal stenosis, Spine surgery

## Abstract

**Background:**

Thoracic ossification of ligamentum flavum (T-OLF), as one of the causes of thoracic myelopathy, is often combined with other spinal disorders. Concurrent lumbar spinal canal stenosis (LCS) is often obscured by symptoms due to T-OLF, leading to difficulty in identifying the origin of these neurological findings. It is common to be misdiagnosed or delayed diagnosis due to the complicated nature. We evaluated the prevalence, distribution, and clinical characteristics of OLF, especially in patients with LCS.

**Methods:**

The authors performed a retrospective analysis of the outcomes of 61 patients who underwent thoracic surgeries performed for symptomatic T-OLF. In all the patients, whole spine lesions were evaluated preoperatively. We examined the factors related to poor outcomes (the recovery rate of the Japanese Orthopedic Association score for thoracic myelopathy is less than 40%) following OLF surgeries. We compared the clinical outcomes according to whether there was concurrent LCS, and determined the optimal surgical approach.

**Results:**

The occurrence of T-OLF increased with age. Forty-six cases (75.4%) were considered to be tandem T-OLF and LCS (LCS group). An advanced age, and concurrent LCS were associated with a poor outcome after the surgery. The LCS group significantly included a greater number of elderly, and more light-weighted patients with Modic change in thoracic spine and a greater sagittal vertical axis, resulting in the lower neurological recovery. Additional lumbar surgery (13cases) effectively improved both the T-JOA and L-JOA scores (from 6.5 ± 2.0 points to 8.0 ± 1.8 points, *p* = 0.0406, and from 14.5 ± 4.7 points to 20.7 ± 2.6 points, *p* = 0.001, respectively) in OLF patients with LCS.

**Conclusions:**

T-OLF was highly associated with other spinal disorders. Poor outcomes in T-OLF surgery could be associated with age and concurrent LCS, and an additional surgery for another lumbar lesion significantly improved neurological findings in T-OLF patients.

## Background

The ligamentum flavum may undergo various pathologic changes, including hypertrophy, calcification, cyst formation, haematoma, and ossification [1, 2]. The ossification of the ligamentum flavum (OLF) is characterized by the replacement of the ligamentum flavum by ectopic new bone. OLF is a rare disease entity that is particularly prevalent among Asians in the thoracic spine. The prevalence of thoracic OLF (T-OLF) in the Japanese population has been reported to be 36% [3].

OLF is well known to be a slowly progressive disease and one of the causes of thoracic myelopathy due to compression of the spinal cord from the posterolateral side [4]. OLF is often combined with other spinal disorders, and misdiagnosis and delayed diagnosis commonly occur [5].

When an OLF patient has severe thoracic myelopathy, concurrent lumbar spinal canal stenosis (LCS) is often obscured by symptoms caused by a thoracic lesion, leading to difficulty in identifying the origin of these neurological findings, especially in the lower limbs. Due to the complicated nature of this condition, research on tandem thoracic and lumbar stenosis is extremely limited to studies with small sample sizes [5, 6]. Predominantly, symptomatic lesions are treated surgically. However, concurrent LCS in T-OLF patients may have adverse effects on the surgical outcomes in patients with T-OLF [6]. Despite the high incidence of combined spinal stenosis, there is no consensus on the optimal surgical approach for this condition.

We focused on tandem T-OLF and other stenosis lesions with high incidence and conducted the present study with the aim of evaluating the prevalence and clinical characteristics of tandem T-OLF and LCS in patients with thoracic myelopathy. We compared the clinical outcomes according to whether there was concurrent LCS in a reasonable number of subjects and investigated the efficacy of additional lumbar decompression surgery post thoracic surgery in T-OLF patients.

## Methods

The current retrospective study was approved by our institutional review board. The authors executed a retrospective analysis of the outcomes of 61 consecutive thoracic surgeries performed in symptomatic OLF patients from May 2015 to May 2019 who were followed up at more than 1 year after surgery. Patients who previously underwent thoracic spine surgery and were younger than 15 years were excluded. There were 21 females and 40 males, with an average age of 61.9 ± 16.2 years (range 20–90 years).

The medical records of these patients were reviewed for demographics including the length of follow-up, duration of symptoms, age, sex, height, body weight, preoperative manual muscle test (MMT) results, and preoperative comorbidities. The preoperative global spinal alignment parameters [7], distribution of OLFs in the whole spine, involved T-OLF levels in which we performed decompression surgery, the presence of ossification of the longitudinal ligament (OPLL) and/or diffuse idiopathic skeletal hyperostosis (DISH) [8], and presence/absence of combined spinal lesions were also recorded in this study.

In principle, we conducted myelography assessments for all these patients before performing the surgeries and evaluated not only the thoracic spine but also the cervical and/or lumbar region. Computed tomography (CT) after myelography (CTM) scans were performed using a multi-slice scanner (Optima CT660, GE Healthcare Japan Inc., Tokyo). A compressive lesion of the spinal cord or cauda equine was recorded [9–11].

**Fig. 1:**
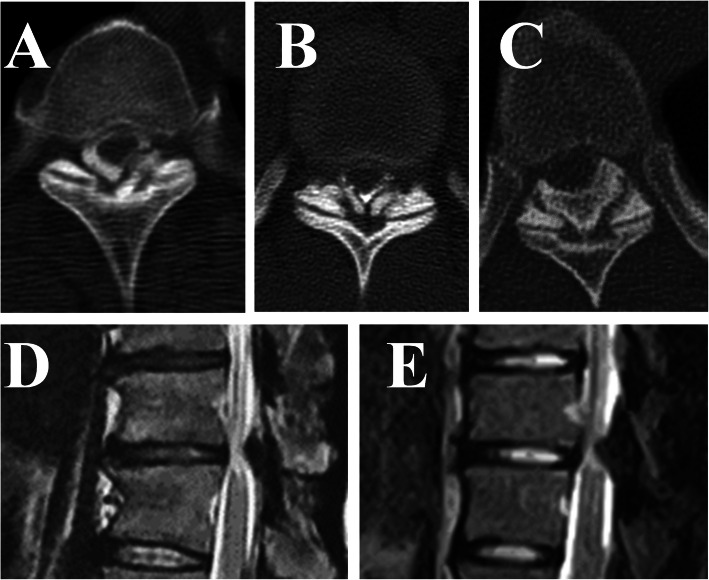
Type of the thoracic OLF configuration. The thoracic OLF was classified into 3 subgroups, unilateral (**a**), bilateral (**b**), and bridged (**c**) types on axial CT scan, and into 2 subgroups, round (**d**), and beak (**e**) types on T2-weighted sagittal MRI. Intramedullary high-intensity areas were shown in both (**d**) and (**e**)

We classified T-OLF cases by type according to the radiologic findings of the symptomatic lesions that required surgical decompression. A T-OLF case was classified as unilateral, bilateral, or bridged (fused) according to the OLF location and type identified by axial CT and classified as a beak or round type according to sagittal magnetic resonance imaging (MRI) [12, 13] (Fig. 1). We also collected data on the changes in involved vertebral body marrow (Modic change) [14] and the intramedullary high-intensity areas (HIA) on T2-weighted MRI (both the sagittal and axial views).

Two spinal surgeons evaluated independently whether OLF was present, and the compressive lesions in the whole spine were assessed with a DICOM viewer. The evaluators reached a consensus by discussion if the results differed. Before the images were reviewed, the testers read the images from the same 20 patients to assess interobserver agreement. The Kappa coefficient for interobserver agreement was 0.76.

While the surgical indications were progressive thoracic myelopathy and leg/dorsal pain, the surgical method was selected on the basis of multiple factors, that is, the compressive pathology, the extent of the degenerative process, intervertebral instability, the sagittal alignment of the thoracic spine, and the patient’s medical conditions [11]. Total or subtotal laminectomy with or without posterior instrumentation was limited to the compressed spinal cord levels, and the OLF was completely resected or floated in all the patients.

In these patients, the operative procedure, operation time, blood loss in the thoracic operation, perioperative complications, and clinical outcomes were investigated. The clinical outcomes were assessed by means of the visual analogue scale (VAS) score for pain or numbness severity from the chest to the toes and the scoring system proposed by the Japanese Orthopaedic Association for the thoracic spine (T-JOA score) and the lumbar spine (L-JOA score). The recovery rate for the T(L)-JOA score was determined by [{postoperative score – preoperative score}/{11(29)-preoperative score}] x 100% [13, 15]. The surgical outcomes were classified into 2 groups: the good outcome group included patients with a recovery rate of the T-JOA score of higher than 40%; the poor outcome group included patients with a recovery rate of less than 40%.

The demographics of the patients included in this study are shown in Table 1. We retrospectively examined the factors related to poor outcomes following T-OLF surgery by performing univariate analyses. We also compared the surgical outcomes, in terms of multiple clinical parameters, between the T-OLF patients with concurrent LCS (LCS group) and those without LCS (non-LCS group). Statistical analysis was performed using Student’s unpaired *t*-tests, Mann-Whitney *U* tests, and Fischer’s exact tests for categorical variables. All the data are expressed as means ± standard deviations (SDs). A *p* value of less than 0.05 was considered to indicate a statistically significant difference.

**Table 1:** Demographics

			*n* = 61
Age, average, average (range), years			61.9 ± 16.1 (20–90)
Sex, female/male, No.			21/40
Duration of disease, average (range), months			17.6 ± 24.2 (1-144)
Follow-up period, average (range), months			32.1 ± 16.0 (12–60)
Preoperative comorbidities, No.		HT	18 (29.5%)
		DM	8 (13.1%)
		IHD	9 (14.8%)
Concurrent spinal canal stenosis, No.		Cervical [Non-C-OPLL/C-OPLL]	23 (37.7%) [11 (18.0%)/12 (19.7%)]
		Thoracic [T-OPLL]	8 (13.1%)
		LCS [Spondylosis/Spondylolisthesis/L-OPLL]	46(75.4%) [26 (42.6%)/18 (29.5%)/2 (3.3%)]
Height, average (range), cm			160.2 ± 10.1 (134–179)
Body weight, average (range), Kg			70.1 ± 19.2 (44.2-103.4)
BMI, average (range), Kg/m2			27.2 ± 6.0 (17.6–52.4)
Dual Energy X-ray Absorptiometry		Lumbar YAM, average (range), %	103.2 ± 17.2 (75–147)
		Femoral YAM, average (range), %	83.7 ± 19.4 (66–137)
MMT, average (range),		Preoperative	3.3 ± 1.4 (0–5)
Operation	Surgical procedure	LAM/PDF, No	44 (72.1%)/17 (27.9%)
	Decompressed OLF levels, average (range), No.		1.5 ± 0.9 (1–4)
	Simultaneous lumbar decompression, No		10 (16.4%)
	Operative time, average (range), min		156.7 ± 97.6 (48–460)
	Blood loss, average (range), mL		284.9 ± 606.8 (1-4090)
Outcomes	T-JOA score, average (range), points	Preoperative	5.9 ± 1.9 (2–10)
		Postoperative	8.0 ± 1.6 (4.5–11)♰
	Recovery rate of T-JOA score, average (range), %		39.2 ± 29.2 (-10-100)
	VAS, average (range), mm	Preoperative	72.2 ± 25.1 (0-100)
		Postoperative	52.7 ± 28.8 (0-100)♰
	Complication, No	Dural tear	3 (4.9%)
		Hematoma	2 (3.3%)
		Vertebral fracture	1 (1.6%)
		SSI	1 (1.6%)
		Enlargement of T-OPLL	1 (1.6%)
Radiological findings	Preoperative global spinal alignment	LL, average (range), degrees	37.9 ± 15.2 (2–67)
		PI, average (range), degrees	46.5 ± 7.6 (22–63)
		SS, average (range), degrees	30.2 ± 10.1 (8–58)
		TLK, average (range), degrees	11.6 ± 12.1 (-8-54)
		TK, average (range), degrees	23.9 ± 11.7 (2–59)
		SVA, average (range), mm	46.6 ± 51.3 (-54-183)
	Local kyphosis, average (range), degrees	Preoperative	9.2 ± 8.4 (0–45)
		Postoperative	11.2 ± 9.4 (0–50)
	CT	DISH, No.	19 (31.1%)
		OLF, Unilateral/Bilateral/Bridged, No.	12 (19.7%)/39 (63.9%)/10 (16.4%)
		Thickness of OLF, average (range), mm	3.6 ± 2.0 (1–10)
		OLF segments, average (range), No.	3.1 ± 2.5 (1–12)
	MRI	OLF, Round/Beak, No.	37 (60.7%)/24 (39.3%)
		Modic change, No.	29 (47.5%)
		HIA, No.	51 (83.6%)

*Abbreviation*: *BMI* body mass index, *MMT* manual muscle test, *T-JOA* the Japanese Orthopaedic Association for thoracic myelopathy, *VAS* visual analog scale, *CT* computed tomography, *MRI* magnetic resonance imaging, *HT* hypertension, *DM* diabetes mellitus, *HL* hyperlipidemia, *IHD* ischemic heart disease, *OPLL* ossification of the longitudinal ligament, *LCS* lumbar spinal canal stenosis, *YAM* young adult mean, *LAM* laminectomy, *PDF* posterior decompression with fusion, *OLF* ossification of the ligamentum flavum, *SSI* surgical site infection, *LL* lumbar lordosis, *PI* pelvic incidence, *SS* sacral slope, *TLK* thoracolumbar kyphosis, *TK* thoracic kyphosis, *SVA* sagittal vertical axis, *DISH* diffuse idiopathic skeletal hyperostosis, *HIA* high-intensity areas

♰*p* < 0.05 when compared to the preoperative status

## Results

A total of 61 patients with myelopathy due to OLF in this study tolerated the surgical procedure well and were followed up for an average of 32.1 months. The duration of symptoms averaged 17.6 months. Statistical analyses revealed that T-OLF was noted at a significantly higher rate among the males (*p* = 0.0005). The preoperative comorbidities included hypertension in 18 cases (29.5%), diabetes mellitus (DM) in 8 cases (13.1%), and ischaemic heart disease in 9 cases (14.8%). The average body mass index (BMI) was 27.2 ± 6.0 kg/m^2^, which is considered to indicate obesity but not osteoporosis (Table 1).

**Fig. 2:**
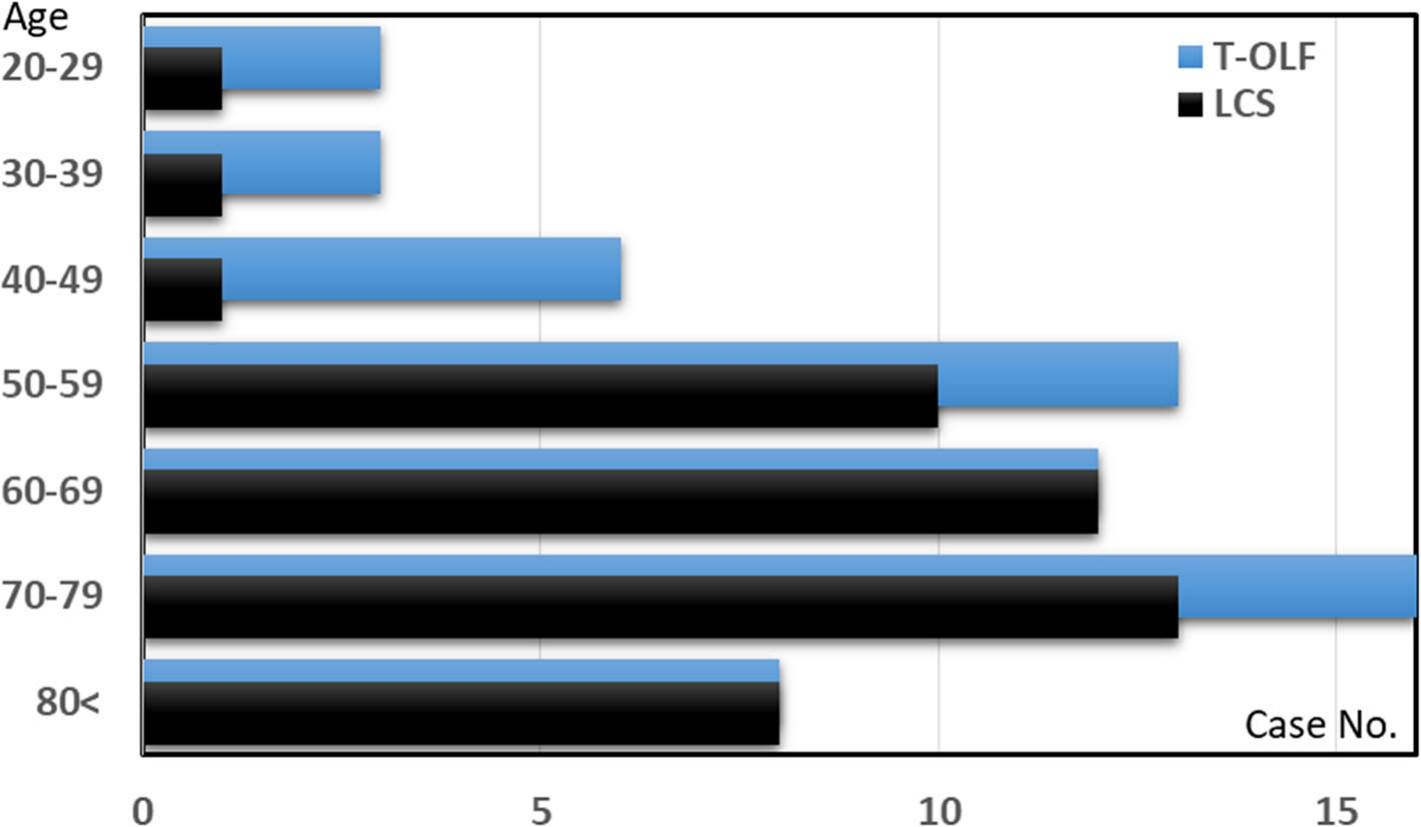
The number of observable thoracic OLF and LCS cases by age group

The myelography and CTM findings indicated that there were 23 cases (37.7%) of radiological cervical spondylotic myelopathy and C-OPLL, 19 cases (31.1%) of DISH, and 46 cases (75.4%) of radiological LCS (LCS group) among the OLF patients who underwent thoracic surgery. Not only the prevalence of T-OLF but also that of coexistent LCS seemed to occur in patients aged mainly between 50 and 80 years, and the occurrence increased with age (Fig. 2). With regard to thoracic surgery, 44 patients (72.1%) underwent laminectomy (LAM) alone, and 17 patients (27.9%) underwent posterior decompression with fusion (PDF). T-OLF patients showed significant improvement both in the T-JOA score (*p* < 0.0001), and in the VAS score (*p* = 0.0008). The mean recovery rate of T-JOA score at the final follow-up was 39.2 ± 29.2%. Perioperative complications occurred in 8 patients (13.1%), and the complicated included dural tears (4.9%), haematomas (3.3%), vertebral fractures (1.6%), and surgical site infections (1.6%). T-OPLL enlargement at the same level was found in 1 patient after the laminectomy procedure, resulting in an additional thoracic spinal fusion surgery.

**Fig. 3:**
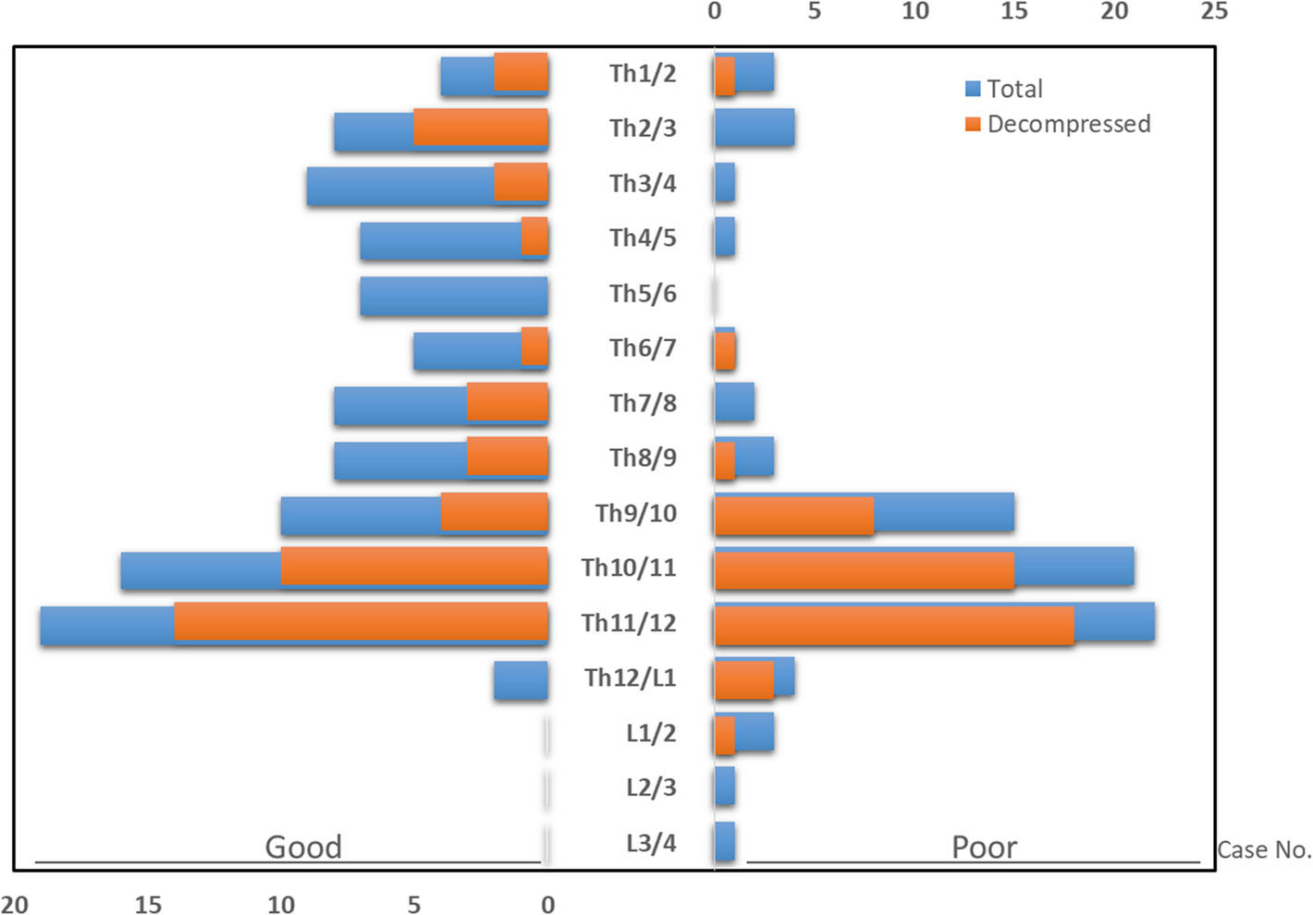
Distribution of OLF in the thoracolumbar region of 61 patients

Apart from the presence of spinal cord compression, the distribution of T-OLFs formed 2 obvious peaks; the highest and second highest peaks were found at Th9-Th12 and Th2-Th5, respectively. Among these OLF segments (an average of 3.1 intervertebral levels, range 1–12 levels), we focused on the compressive OLF segment in which we actually performed surgery (an average of 1.5 intervertebral levels, range 1–4 levels) (Table 1). The most commonly affected and decompressed segment was the Th11/12 intervertebral body level (41 cases, 67.2%), followed by the Th10/11 level (25 cases, 41.0%) (Fig. 3).

The surgical results were good for 31 patients and poor for 30 patients. According to univariate analyses of surgical outcomes, an advanced age, a short stature, and the presence of concurrent lumbar stenosis were associated with poor outcomes after surgery (65.9 ± 15.2 years vs. 57.8 ± 16.3 years, *p* = 0.049, 157.1 ± 11.0 cm vs. 163.4 ± 8.2 cm, *p* = 0.0139, and 27 cases vs. 19 cases, *p* = 0.0314, respectively) (Table 2).

**Table 2:** Comparison between the cases with poor clinical outcomes and those with good outcomes

			Poor *n* = 31	Good *n* = 30
Age, average, average (range), years			65.9 ± 15.2 (22–90)	57.8 ± 16.3 (20–81)*
Sex, female/male, No.			13/18	8/22
Duration of disease, average (range), months			20.4 ± 29.2 (1-144)	14.7 ± 17.6 (1–72)
Concurrent spinal canal stenosis, No.		Cervical [Non-C-OPLL/C-OPLL]	8 (25.8%)[4/4]	15 (50.0%)[7/8]
		LCS [Spondylosis/Spondylolisthesis/L-OPLL]	27 (87.1%)[14/12/1]	19 (63.3%)[12/6/1]*
Height, average (range), cm			157.1 ± 11.0 (134–176)	163.4 ± 8.2 (148–179)*
Body weight, average (range), Kg			65.8 ± 14.7 (44.2-103.4)	74.4 ± 22.4 (46.7-100.7)
Dual Energy X-ray Absorptiometry		Lumbar YAM, average (range), %	101.9 ± 14.7 (75–116)	104.3 ± 19.9 (77–147)
		Femoral YAM, average (range), %	90.0 ± 24.8 (62–137)	77.3 ± 9.6 (66–95)
MMT, average (range),		Preoperative	3.1 ± 1.4 (0–5)	3.5 ± 1.4 (0–5)
Operation	Surgical procedure	LAM/PDF, No	23/8	21/9
	Decompressed OLF levels, average (range), No.		1.5 ± 0.9 (1–4)	1.5 ± 0.9 (1–4)
	Operative time, average (range), min		150.6 ± 90.3 (48–436)	163.0 ± 105.8 (56–460)
	Blood loss, average (range), mL		259.2 ± 726.4 (1-4090)	311.5 ± 463.2 (1-1725)
Outcomes	Recovery rate of T-JOA score, average (range), %		15.8 ± 13.4 (-10-36.4)	63.4 ± 19.9 (40–100)*
	VAS, average (range), mm	Preoperative	77.2 ± 21.4 (30–100)	66.1 ± 28.4 (0-100)
		Postoperative	56.6 ± 27.0 (10–100)♰	47.9 ± 30.9 (10–100)
	Complication, No		4 (12.9%) [Dural tear, Hematoma, Vertebral fracture, SSI = 1]	4 (13.3%) [Dural tear = 2, Hematoma, Enlargement of T-OPLL = 1]
Radiological findings	Local kyphosis, average (range), degrees	Preoperative	8.9 ± 10.2 (0–45)	9.4 ± 6.2 (1–32)
		Postoperative	10.6 ± 11.0 (0–50)	11.8 ± 7.7 (2–37)
	CT	DISH, No.	9 (29.0%)	10 (33.3%)
		OLF, Unilateral/Bilateral/Bridged, No.	7/20/4	5/19/6
		Thickness of OLF, average (range), mm	3.2 ± 1.6 (1-6.7)	4.0 ± 2.3 (1–10)
	MRI	OLF, Round/Beak, No.	18/13	19/11
		Modic change, No.	13 (41.9%)	11 (36.7%)
		HIA, No.	24 (77.4%)	27 (90.0%)

*Abbreviation*: *MMT* manual muscle test, *T-JOA* the Japanese Orthopaedic Association for thoracic myelopathy, *VAS* visual analog scale, *CT* computed tomography, *MRI* magnetic resonance imaging, *OPLL* ossification of the longitudinal ligament, *LCS* lumbar spinal canal stenosis, *YAM* young adult mean, *LAM* laminectomy, *PDF* posterior decompression with fusion, *OLF* ossification of the ligamentum flavum, *SSI* surgical site infection, *DISH*, diffuse idiopathic skeletal hyperostosis, *HIA* high-intensity areas

*p < 0.05 when compared to the poor outcome group

♰*p* < 0.05 when compared to the preoperative status

Comparing the non-LCS group (*n* = 15) and the LCS group (*n* = 46), the LCS group included a significantly larger number of elderly people, more lightweight patients with Modic change and a greater sagittal vertical axis (SVA) (48.1 ± 17.3 years vs. 66.4 ± 13.1 years, *p* < 0.0001, and 83.0 ± 27.3 kg vs. 65.9 ± 13.6 kg, *p* = 0.0021, Modic change; 3 cases vs. 26 cases, *p* = 0.0139, and 18.5 ± 42.1 mm vs. 55.6 ± 51.1 mm, *p* = 0.0172, respectively), while the coexistence rate of C-OPLL and the thickness of the involved OLF were smaller than those in the non-LCS group. In the LCS group, the mean preoperative MMT grade and the mean postoperative T-JOA score were significantly lower than those in the non-LCS group (4.0 ± 0.9 vs. 3.1 ± 1.5, *p* = 0.0241, and 8.7 ± 1.4 points vs. 7.8 ± 1.6 points, *p* = 0.0482, respectively). While the VAS score significantly decreased in the LCS group (*p* = 0.0018), the mean recovery rate of the T-JOA score was relatively inferior to that of the non-LCS group (47.9 ± 29.2% vs. 36.4 ± 29.0%, *p* = 0.1887) (Table 3).

**Table 3:** Comparison between the cases without LCS and those with LCS

			Non-LCS *n* = 15	LCS *n* = 46
Age, average, average (range), years			48.1 ± 17.3 (22–78)	66.4 ± 13.1 (27–90)*
Sex, female/male, No.			5/10	16/30
Concurrent spinal canal stenosis, No.		Cervical [Non-C-OPLL/C-OPLL]	8 (53.3%) [2/6]	15 (32.6%) [9/6]
Height, average (range), cm			164.0 ± 10.2 (148–179)	159.0 ± 9.9 (134–176)
Body weight, average (range), Kg			83.0 ± 27.3 (56.3-100.7)	65.9 ± 13.6 (44.2-103.4)*
Dual Energy X-ray Absorptiometry		Lumbar YAM, average (range), %	104.0 ± 15.6 (93–115)	103.1 ± 17.9 (75–147)
		Femoral YAM, average (range), %	90.5 ± 27.6 (71–110)	82.8 ± 19.2 (62–137)
MMT, average (range),		Preoperative	4.0 ± 0.9 (2–5)	3.1 ± 1.5 (0–5)*
Operation	Surgical procedure	LAM/PDF, No	13/2	31/15
	Decompressed OLF levels, average (range), No.		1.5 ± 1.1 (1–4)	1.5 ± 0.8 (1–4)
Outcomes	T-JOA score, average (range), points	Preoperative	6.3 ± 1.8 (2-8.5)	5.7 ± 2.0 (2–10)
		Postoperative	8.7 ± 1.4 (5.5–11)♰	7.8 ± 1.6 (4.5–11)♰*
	Recovery rate of T-JOA score, average (range), %		47.9 ± 29.2 (0-100)	36.4 ± 29.0 (-10-100)
	VAS, average (range), mm	Preoperative	60.5 ± 27.6 (0-100)	75.7 ± 23.7 (30–100)
		Postoperative	45.5 ± 29.5 (0–90)	55.2 ± 28.6 (10–100)♰
Radiological findings	Preoperative global spinal alignment	LL, average (range), degrees	42.8 ± 15.7 (11–65)	36.3 ± 14.8 (2–67)
		PI, average (range), degrees	46.3 ± 5.6 (11–52)	46.6 ± 8.2 (22–63)
		SS, average (range), degrees	30.9 ± 9.4 (8–58)	30.0 ± 10.4 (8–58)
		TLK, average (range), degrees	12.5 ± 7.6 (-2-31)	11.3 ± 13.3 (-8-54)
		TK, average (range), degrees	28.4 ± 11.8 (13–59)	22.4 ± 11.4 (2–50)
		SVA, average (range), mm	18.5 ± 42.1 (-54-99)	55.6 ± 51.1 (-25-183)*
	CT	DISH, No.	3 (20%)	16 (34.8%)
		OLF, Unilateral/Bilateral/Bridged, No.	1/8/6	11/31/4*
		Thickness of OLF, average (range), mm	4.8 ± 2.4 (2.4–10)	3.2 ± 1.7 (1-8.9)*
	MRI	OLF, Round/Beak, No.	8/7	29/17
		Modic change, No.	3 (20%)	26 (56.5%)*
		HIA, No.	11 (73.3%)	40 (87.0%)

*Abbreviation*: *MMT* manual muscle test, *T-JOA* the Japanese Orthopaedic Association for thoracic myelopathy, *VAS* visual analog scale, *CT* computed tomography, *MRI* magnetic resonance imaging, *OPLL* ossification of the longitudinal ligament, *LCS* lumbar spinal canal stenosis, *YAM* young adult mean, *LAM* laminectomy, *PDF* posterior decompression with fusion, *OLF* ossification of the ligamentum flavum, *LL* lumbar lordosis, *PI* pelvic incidence, *SS* sacral slope, *TLK* thoracolumbar kyphosis, *TK* thoracic kyphosis, *SVA* sagittal vertical axis, *DISH* diffuse idiopathic skeletal hyperostosis, *HIA* high-intensity areas

*p < 0.05 when compared to the non-LCS group

♰*p* < 0.05 when compared to the preoperative status

We investigated to what extent performing a lumbar operation in addition to T-OLF surgery affects the postoperative outcomes of tandem T-OLF and lumbar stenosis patients. We divided 46 T-OLF patients with concurrent LCS into two groups according to whether they underwent additional lumbar operations. The mean recovery rate of the T-JOA score at the final follow-up and ΔVAS score did not differ significantly in the tandem T-OLF and LCS patients based on whether they did or did not undergo additional lumbar surgery. Nevertheless, in the additional lumbar surgery group, the T-JOA score before lumbar surgery was 6.5 ± 2.0 points, but it significantly improved to 8.0 ± 1.8 points after lumbar surgery (*p* = 0.0406). In addition, the recovery rate of the L-JOA score itself (41.4 ± 12.5%) seemed satisfactory (Table 4). These results suggest that additional lumbar surgery effectively improved both the T-JOA and L-JOA scores in OLF patients with symptomatic LCS.

**Table 4:** Comparison between the cases without lumbar surgery and those with lumbar surgery

			Only thoracic OLF operation *n* = 33	Additional lumbar operation *n* = 13
Age, average, average (range), years			66.3 ± 12.8 (27–90)	66.5 ± 14.2 (33–81)
Sex, female/male, No.			14/19	2/11
Duration of disease, average (range), months			18.5 ± 29.2 (1-144)	20.2 ± 21.7 (3–72)
MMT, average (range),		Preoperative	3.1 ± 1.5 (0–5)	3.1 ± 1.5 (0–5)
Operation	Surgical procedure	LAM/PDF, No	22/11	9/4
	Decompressed OLF levels, average (range), No.		1.5 ± 0.8 (1–4)	1.4 ± 0.7 (1–3)
	Operative time, average (range), min		133.9 ± 90.8 (48–460)	215.5 ± 98.7 (60–436)*
	Blood loss, average (range), mL		127.2 ± 185.1 (1-893)	496.8 ± 1097.9 (1-4090)
Outcomes	T-JOA score, average (range), points	Prethoracic operative	5.5 ± 1.9 (2–10)	6.3 ± 2.0 (3-9.5)
		Prelumbar operative	-	6.5 ± 2.0 (3.5–9.5)
		Postoperative	7.7 ± 1.5 (5.5–11)♰	8.0 ± 1.8 (4.5–11)♰$
	Recovery rate of T-JOA score, average (range), %		35.7 ± 29.6 (-10-88.9)	38.1 ± 28.6 (0-100)
	L-JOA score, average (range), points	Preoperative	-	14.5 ± 4.7 (9–24)
		Postoperative	-	20.7 ± 2.6 (16–26)$
	Recovery rate of L-JOA score, average (range), %		-	41.4 ± 12.5 (20.0-68.4)
	VAS, average (range), mm	Preoperative	76.5 ± 23.9 (30–100)	73.6 ± 24.2 (30–100)
		Postoperative	56.5 ± 32.1 (10–100)♰	52.5 ± 20.8 (0–70)♰

*Abbreviation*: *MMT* manual muscle test, *T-JOA* the Japanese Orthopaedic Association for thoracic myelopathy, *L-JOA* the Japanese Orthopaedic Association for Lumbar spinal canal stenosis, *VAS* visual analog scale, *LAM* laminectomy, *PDF* posterior decompression with fusion, *OLF* ossification of the ligamentum flavum

*p < 0.05 when compared to the only thoracic OLF operation group

♰*p* < 0.05 when compared to the prethoracic operative status

$*p* < 0.05 when compared to the prelumbar operative status

During the follow-up period, staged lumbar decompression surgery for LCS was performed in 3 patients (4.9%) due to the progression of leg pain and/or the deterioration of MMT after thoracic decompression. The mean period between thoracic and lumbar lesion surgery was 13.0 months. The neurologic symptoms were relieved significantly in all three patients after the lumbar surgeries. The mean T-JOA score improved from 4.3 points to 6.2 points after the additional staged lumbar surgery (*p* = 0.1365). The present study included 10 patients (16.4%) who underwent single-stage simultaneous thoracic and lumbar surgery. The mean recovery rate of the T-JOA score at the final follow-up was 39.8 ± 30.0%. This rate could not significantly get over that in the staged surgery patients (39.8 ± 30.0% vs. 32.3 ± 28.4%, *p* = 0.7124).

## Discussion

First, primary thoracic spinal stenosis is rare [5]. The formation of T-OLF is strongly considered due to degenerative wear and tear, as well as external triggers such as biomechanical stress due to posttraumatic ossification [16, 17]. There are reports showing that OLF manifestations are related to DM, heavy manual labour in males, high BMI values in females, increased bone density, and DISH [18], and these findings are in accordance with the demographic results in our study. Thus, T-OLF coincides with spinal osteoarthritis and spondylosis deformans, leading to a high incidence of tandem T-OLF and other stenosis lesions in the cervical or lumbar area, sometimes with the ossification of ligaments. Cases with the ossification of ligaments have a tendency to develop into tandem spinal stenosis (TSS) [10]; therefore, T-OLF seems to be no exception, as T-OLF is frequently found in C-OPLL patients [3]. Although spondylotic process is mainly observed in the lower lumbar spine of T-OLF patients, the incidence of lumbar ossified lesions (L-OLF or L-OPLL) is relatively low (5 cases, 8.2%). In the present study, up to 75.4% of thoracic myelopathic patients who underwent T-OLF surgeries had LCS, which was higher than that reported in a study in cervical myelopathic patients (57.9%) [10] but consistent with that reported in a previous study about thoracic myelopathy [19].

Patients with compressive myelopathy due to especially lower thoracic lesions often present with pain, numbness, or motor disturbance in the lower limbs and neurologic claudication, which is consistent with the symptoms of lumbar radiculopathy or cauda equine lesions. Actually, with or without LCS, most of the T-OLF patients in this study suffered from leg pain, numbness, or dysfunction. Spasticity of the lower limbs with hyperreflexia is often masked by cauda equine or lumbar nerve root disorders [6, 20]. In patients with complicated characteristics, it is difficult to diagnose thoracic pathologies. Despite the rate of combined spinal stenosis being high, there have been few reports describing the clinical course of tandem thoracic and lumbar stenosis [5, 6, 20]. The present study includes a reasonable number of tandem T-OLF and LCS subjects and discusses several aspects of these peculiar cases.

Some reports have mentioned that a short preoperative symptom duration, a single-level lesion, and the unilateral type were favourable prognostic factors, while the bridged (fused) type, the beak type, intramedullary HIA on T2-weighted images, concomitant intervertebral degeneration (Modic change), and the presence of a ventral compressive lesion were poor prognostic factors for T-OLF surgery [11, 12, 21]. Contrary to our expectations, we revealed that these factors were not statistically associated, but that age, a short stature, and coexistent LCS were related to a poor prognosis. Considering that the number of patients with cervical or upper thoracic lesions was smaller in the poor outcome group than in the good outcome group (Fig. 3), the dynamic biomechanical factor based on their predilection in the lower thoracic spine could be attributed to progressive thoracic myelopathy through compression of the spinal cord.

Second, compared with the non-LCS patients, the patients with tandem T-OLF and LCS in this study exhibited an older age, a lower preoperative MMT grade, a greater SVA, and degenerative Modic changes in thoracic lesions, resulting in lower postoperative T-JOA scores. Interestingly, the younger non-LCS group exhibited a higher coexistence rate of C-OPLL. The early formation of tandem ossifications, early detection, and younger age at the time of T-OLF surgery could partially explain the good T-JOA scores found in the non-LCS group. An older age might be not only a determining factor of tandem T-OLF/LCS occurrence (Fig. 2) [18] but also one of the most influential factors of neurological improvement in patients with T-OLF. Eskander et al. reported that age increases the risk of major and minor complications, regardless of the surgical algorithm used to manage tandem stenosis lesions [22]. Other reports have concluded that the coexistence of LCS in T-OLF patients has adverse effects on their surgical outcomes, and vice versa [5, 6]. These findings seem to support our conclusion that the prognosis for T-OLF paralleled that of isolated lumbar stenosis, rather than the preoperative symptom duration, the surgical procedures, the local T-OLF size, morphology, the number of affected OLF lesions, or presence of radiographic cervical stenosis. Although the causal relationship between compressive lesions at levels below the operative site and the appearance of symptoms could not be confirmed in the present study, the presence of LCS itself might partially augment the risk of thoracic cord compression, with this lumbar stenosis lesion serving as a fulcrum according to the lever principle; in addition, asymptomatic LCS might naturally progress, leading to the development of lumbar-related symptoms within a few years after thoracic surgery.

The question of how to treat these elderly patients with tandem combined thoracic and lumbar stenosis who have neurological deficits in the lower limbs remains. As a rule, in the operative sequence for tandem T-OLF and LCS, the most clinically symptomatic area should be decompressed first. Radiological LCS itself does not always affect the clinical symptoms. If neurological symptoms originate equally from both the thoracic and lumbar spine and there are severe radiological findings, both lesions require surgical decompression. We generally addressed the thoracic spine first. Thoracic decompression may improve lumbar symptoms because the lumbar neural fibres may also be under compression by the processes T-OLF formation. Among these cases, in cases without severe systemic problems, simultaneous thoracic and lumbar decompression is sometimes performed after an adequate and careful explanation of its invasiveness to the patient.

We further investigated the clinical outcomes of lumbar decompression in OLF patients to clarify the impact of concurrent LCS. A total of 21.3% of the T-OLF patients required additional lumbar surgery. Although the recovery rate of the T-JOA score did not reach significance with or without lumbar decompression for LCS, we determined the effectiveness of additional lumbar decompression in cases of symptomatic tandem T-OLF and LCS after thoracic treatment. It remains unknown which procedure, two-stage or single-stage surgery, is more effective for achieving postoperative neurologic improvements in T-OLF patients with symptomatic LCS. The degree of neurological recovery after simultaneous single-stage surgery in tandem T-OLF and LCS cases was also equal to that after staged surgery. This result suggested that lumbar operations can be staged if the single-stage surgery is considered to be too invasive for the patient. Careful observation is needed after the first thoracic surgery for these patients to undergo an additional lumbar surgery at the appropriate time.

As one of the limitations, this analysis was based on a simple comparison of the widely accepted T-JOA score in evaluating thoracic surgical outcomes, and we did not compare the T-JOA score to other tests. As shown in Tables 2 and 3, the improvement in the VAS score did not always reflect that in the T-JOA score. These results indicated that good improvement in the T-JOA score could reflect improvement in the neurological functional status, whereas subjective symptoms were expressed in the VAS score. This study has several other limitations, including the following: (1) the patient baseline was not controlled, and the number of subgroup analysis was limited; (2) this was a retrospective investigation; and (3) the assessment of the correlation between the dynamic factor in OLF levels and clinical outcomes was inadequate. As mentioned above, these limitations could be partially ascribed to the challenges of assessing and treating the neurological symptoms related to OLF and those of performing biomechanical evaluations in the thoracic spinal system, which has a limited range of segmental motion. Other sophisticated evaluation methods and a large number of prospective data need to be collected in the future to study the clinical outcomes of OLF cases in more detail.

Given these results, T-OLF may be related to an individual’s age and lifestyle, and is likely accompanied by other spinal stenosis lesions. The identification of the predominant lesion and the interpretation of clinical outcomes seems to be controversial and difficult in T-OLF cases. We focused on tandem T-OLF and LCS, which are highly prevalent. The prognosis for patients with T-OLF and concurrent LCS was worse than that for patients without LCS. The present study results concluded that the severity of lumbar lesions could have great influence on neurological improvement after thoracic surgery for OLF, and an additional surgery for another lumbar lesion could significantly improve the neurological outcomes. Therefore, surgical management in these tandem stenosis should be tailored to the patient’s age and general condition, whether the procedures are performed simultaneously or in stages, as noted previously [10, 11].

## Conclusions

T-OLF was highly associated with other spinal disorders. Ageing and the coexistence of lumbar lesions influenced the recovery process following surgery for thoracic myelopathy due to T-OLF. Additional lumbar decompression appears to be warranted in situations where some symptoms persist after thoracic decompression.

## Data Availability

The datasets used and/or analyzed during the current study are available from the corresponding author on reasonable request.

## Abbreviations

OLF: Ossification of the ligamentum flavum
T-OLF: Thoracic OLF
LCS: Lumbar spinal canal stenosis
MMT: Manual muscle test
OPLL: Ossification of the longitudinal ligament
DISH: Diffuse idiopathic skeletal hyperostosis
CT: Computed tomography
CTM: CT myelography
MRI: Magnetic resonance imaging
HIA: High-intensity areas
VAS: Visual analog scale
T-JOA: The Japanese Orthopaedic Association for thoracic myelopathy
L-JOA: The Japanese Orthopaedic Association for Lumbar spinal canal stenosis
SD: Standard deviation
DM: Diabetes mellitus
BMI: Body mass index
LAM: Laminectomy
PDF: Posterior decompression with fusion
SVA: Sagittal vertical axis
TSS: Tandem spinal stenosis
